# Prevalence of periodontal diseases: Latin America and the Caribbean Consensus 2024

**DOI:** 10.1590/1807-3107bor-2024.vol38.0116

**Published:** 2024-11-22

**Authors:** Paola CARVAJAL, Fernanda Campos de Almeida CARRER, Mariana Lopes GALANTE, Rolando VERNAL, Cristina Barboza SOLIS

**Affiliations:** (a) Universidad de Chile, School of Dentistry, Department of Conservative Dentistry, Santiago, Chile.; (b) Universidade de São Paulo – USP, School of Dentistry, Department of Community Dentistry, São Paulo, SP, Brazil.; (c) Universidade de São Paulo – USP, School of Dentistry, Department of Community Dentistry, São Paulo, SP, Brazil.; (d) Universidad de Chile, School of Dentistry, Periodontal Biology Laboratory, Santiago, Chile.; (e) Universidad de Costa Rica, School of Dentistry, Depatment of Epidemiology, San Jose, Costa Rica.

**Keywords:** Prevalence, Periodontitis, Gingivitis, Latin America, Caribbean Region, Adults, Adolescent

## Abstract

The aim of this review was to update knowledge about the prevalence of periodontitis in Latin America and the Caribbean. A critical review of was performed of all cross-sectional or cohort studies selected, pertaining to the region, and thirty-five studies conducted in 12 countries were selected. The countries with nationally representative studies were Brazil, Chile, Colombia, and Uruguay. The prevalence of periodontal disease or need for periodontal treatment varied between the different studies and countries depending on the age group, the methodology used, and the case definition. The prevalence of severe periodontitis aged between 5.8% and 49.7% in adults. In adolescents, the prevalence of moderate to severe periodontitis was 15.3%. Furthermore, a high prevalence of gingival bleeding in adolescents was reported. When analyzing the studies that used the Community Periodontal Index (CPI), Centers for Diseases Control and American Academy Periodontology (CDC/AAP) case definition, it was observed that as the age of the individuals analyzed increased, the prevalence of periodontal disease also increased. Whereas this rereview revealed that although the number of regional and nationally representative studies that analyzed the prevalence of periodontitis has risen in recent years, their methodological heterogeneity prevents global conclusions to be drawn concerning the region. Therefore, this ratifies the need to generate alliances between countries with the purpose of joining individual efforts to achieve collective goals which, among other objectives, will translate into conducting multicenter studies. These studies would allow description and monitoring of the epidemiological behavior of periodontitis in Latin America and the Caribbean.

## Introduction

Periodontitis is considered a public health problem given its high prevalence, significant socioeconomic impact since it compromises the quality of life and systemic health of individuals.^1-3^ Indeed, the high prevalence of severe periodontitis contributes to the global burden of chronic non-communicable diseases.^4^

Although the condition has been intensively studied in high-income countries, there is a scarcity of epidemiological studies analyzing the prevalence of periodontitis in low- and middle-income countries.^5^ This applies particularly ton Latin American and Caribbean countries, where there are few data on the prevalence of periodontitis, and methodologies and case definitions have not yet been standardized.^6-8^ Nevertheless in Latin America, these few studies have reported high prevalence of periodontitis in urban and isolated regions, a situation that is strongly determined by factors such as individuals’ education level, socioeconomic status, and income.^6,7,9^ For instance, in 2023 a systematic review was carried out, covering 15 studies with dentate people, conducted in Latin American or Caribbean countries between 2010 and 2021. However, these studies assessed specific populations without any national representation.^5^ In 2015, two critical reviews reported that studies on periodontitis prevalence with national representation were scarce in Latin America.^6,7^ Furthermore, these studies were conducted with substantial methodological heterogeneity, compromising the comparison between countries and regions. In spite of this, they reveal that periodontal attachment loss was more prevalent in Latin America than in the United States and Europe.^6,7^

Therefore, it is necessary to carry out an updated review of the evidence available, including that which was recently published, in order to have a complete overview of the prevalence of periodontitis in Latin America and thus have helpful information for appropriate decision-making on periodontal health. This study summarizes and discusses the scientific articles published until 2023, which report on the prevalence of periodontitis in adolescents and adults in Latin American and Caribbean countries.

## Methods

### Information sources and search strategy

One author (PC) performed the electronic search in PubMed and LILACS (Latin-American Scientific Literature in Health Sciences) databases. The following algorithm built with MeSH terms was used for the PubMed search: “(periodontitis OR gingivitis [MeSH] OR ‘chronic periodontitis’ OR (periodontal diseases [MeSH]) OR ‘attachment loss’ OR pocket) AND (prevalence [MeSH] OR epidemiology [MeSH]) AND (‘South America’ OR Caribbean OR “Latin America”)”. For LILACS, the following algorithm was used: “ab:((periodontitis OR gingivitis OR ‘periodontitis crónica’ OR ‘enfermedad periodontal’ OR ‘pérdida inserción periodontal’ OR ‘bolsa periodontal’) AND (prevalence OR epidemiología)) AND (db:(“LILACS”))”. Furthermore, the same author performed a manual search specifying the name of each Latin American and Caribbean country in the journals from which the initial studies were selected.

### Type of studies and inclusion criteria

The eligibility criteria were cross-sectional or cohort epidemiological studies that reported the prevalence of periodontal diseases, as clinical attachment loss (CAL), gingivitis, or periodontitis, in adolescents and/or adults until December 2023, without limit on publication date, no language restriction, with random sampling, and representative of at least one locality, city, region, or country in Latin America or the Caribbean. Moreover, secondary data analysis studies were included when they provided a different result from the original research.

### Exclusion criteria

Since the interest of the present study was to identify the general population prevalence, studies that reported it in specific populations (including indigenous people, pregnant women, a population with a particular disease or condition, beneficiaries of a health center, or attendees of a specific university clinic) were excluded. Additionally, literature reviews, studies on self-report of periodontal diseases, and studies in which the case definition used was not specified or was only based on the gingival index were excluded.

### Data selection, extraction, and presentation

Preliminarily, relevant articles were selected screening a title and abstract, thus excluding those that were not relevant according to the inclusion and exclusion criteria detailed above. The full text of all eligible studies were read,, and the reasons for exclusion were presented in detail. The final agreement to exclude articles was made collectively during meetings of the research team.

The studies selected were organized in Tables by age group (adolescents and adults), describing the study characteristics and their main results. Information included: first author’s name, publication year, city and country were the analyses were performed, inclusion or exclusion criteria, design, sample size, periodontal examination protocol, age range of participants, periodontal criteria used (periodontal case definition), global prevalence, and prevalence by sex.

In order to standardize and systematize the present study findings, the most used periodontal indicator, Community Periodontal Index (CPI) proposed by the World Health Organization (WHO) for population epidemiological studies,^10^ was used as a reference to ensure comparison between countries. This was accomplished by categorizing the results into CPI > 2 and CPI = 4; a person having a CPI > 2 when presenting a probing depth (PD) > 3 mm in at least one sextant and a person having a CPI = 4 when presenting a PD > 5.5 mm in at least one sextant. In addition, results from studies using the Centers for Diseases Control and American Academy Periodontology (CDC/AAP) case definition were independently presented.^11,12^ Mild periodontitis was defined as ≥2 interproximal sites with CAL ≥ 3 mm and ≥ 2 interproximal sites with PD ≥ 4 mm (not on the same tooth) or one site with PD ≥ 5 mm. Moderate periodontitis was defined as ≥ 2 interproximal sites with CAL ≥ 4 mm (not on the same tooth) or ≥ 2 interproximal sites with PD ≥ 5 mm (also not on the same tooth). Severe periodontitis was defined as ≥ 2 interproximal sites with CAL ≥6 mm (not on the same tooth) and ≥ 1 interproximal site with PD ≥ 5 mm.

## Results

### Study selection

**Figure 1:**
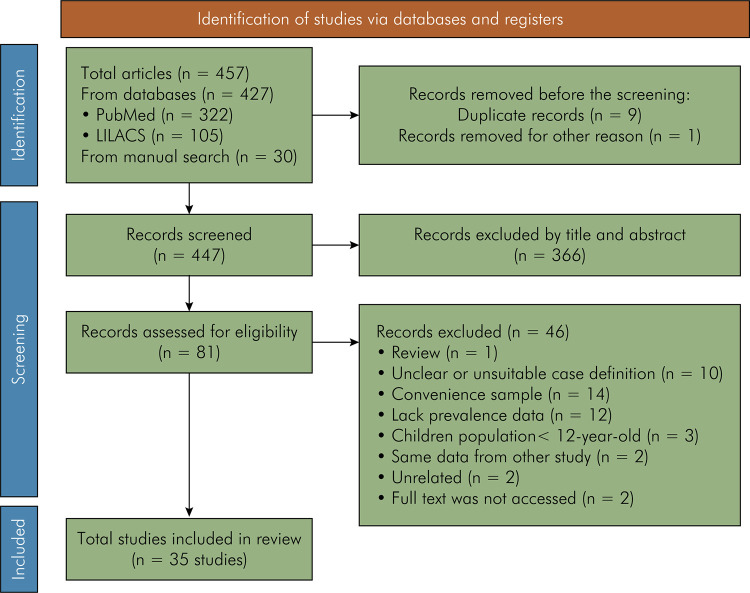
Flow diagram of literature search and selection criteria, based on the PRISMA 2020 statement.63

The initial electronic search strategy yielded 457 articles, 322 from PubMed, 105 from LILACS, and 30 from manual search (Figure 1). These articles were written in English, Spanish, or Portuguese. After removing duplicates, an additional article was discarded because its abstract was inaccessible. Then, 447 articles were identified by screening the title and abstract g, and 366 were removed because they did not comply with the inclusion criteria. The full-text assessment resulted in the inclusion of 81 articles. Of these, 46 articles were excluded; and the reasons for exclusion are summarized in Figure 1 and Table 1. Ultimately, 35 articles were included in the present study, ,of which ,17 reported periodontitis prevalence results in adults,^13-29^ 14 in adolescents,^30-43^ and 4 in both populations.^44-47^

**Table 1:** Excluded studies after full text revision and with their reasons (n = 46).

Authors, year	Country, City	DOI or link	Exclusion criteria
Alonge; Narendran (1999)	St. Vincent and The Grenadines	https://pubmed.ncbi.nlm.nih.gov/11372121/	8
Andrade; Rapp (2002)	District of Barra / Rio Vermelho, Brazil	https://pubmed.ncbi.nlm.nih.gov/12670092/	5
Bonanato et al. (2010)	Belo Horizonte, Minas Gerais, Brazil	https://pubmed.ncbi.nlm.nih.gov/20589245/	2
Carvajal et al. (2016)	South America	doi: 10.1590/1678-775720160178.	2
Castrejón-Pérez et al. (2017)	Mexico	doi: 10.1093/gerona/glw201	3
Chiapinotto et al. (2012)	Brazil, Pelotas	doi: 10.1111/jphd.12001	5
Collins et al. (2019)	Caribbean. Jamaica, Dominican Republic, and Puerto Rico.	doi: 10.1186/s12903-019-0931-1	4
Cortelli et al. (2008)	Brazil	doi: https://doi.org/10.14295/bds.2008.v11i2.448.	3
Cyrino et al. (2011)	Belo Horizonte, Brazil	doi: 10.1902/jop.2011.110015.	3
Duque (2016)	Latin America	http://dx.doi.org/10.1016/j.piro.2016.07.005.	1
Elías-Boneta et al. (2017)	San Juan, Puerto Rico	https://pubmed.ncbi.nlm.nih.gov/28915302/	3
Elías-Boneta et al. (2018)	Caribbean. Jamaica, Dominican Republic, and Puerto Rico.	https://pubmed.ncbi.nlm.nih.gov/29905923/	2
Feldens et al. (2006)	Canoas, Brazil	https://pubmed.ncbi.nlm.nih.gov/16734306/	5
Giacaman et al. (2015)	Maule, Chile	https://pubmed.ncbi.nlm.nih.gov/26108477/	4
Giacaman et al. (2018)	Maule, Chile	doi: 10.22605/RRH4312.	6
Gianopoulos et al. (2014)	Santa Ana, Nicaragua	doi: 10.1111/idh.12043	3
Haas et al. (2015)	Brazil	doi: 10.1590/1980-5497201500020018	4
Haas et al. (2019)	Brazil, Porto Alegre	doi.org/10.1590/1807-3107bor-2019.vol33.0036	2
Ismail; Szpunar (1990)	Mexican Americans, Cuban Americans, and Puerto Ricans	doi: 10.2105/ajph.80.suppl.66.	7
Lock et al. (2020)	Brazil, Porto Alegre	doi: 10.1111/jre.12743	2
Lopez et al. (2002)	Santiago, Chile	doi: 10.1034/j.1600-0765.2002.01377.x.	2
Lorenzo-Erro (2022)	Uruguay	doi: 10.54589/aol.35/3/178.	2
Lorenzo-Erro (2018)	Uruguay	doi: 10.1590/1807-3107bor-2018.vol32.0062.	7
Maltz et al. (2001)	Porto Alegre, Brazil	doi: 10.1007/s007840100122.	4
Medeiros et al. (2022)	Brazil	doi: 10.1002/JPER.21-0433.	4
Medina-Solís et al. (2014)	Mexico	doi: 10.3390/ijerph110303169	4
Moreira et al. 2009	Southeastern Sao Paulo State, Brazil	doi: 10.1590/s1678-77572009000300008.	4
Moreno de Calafell; Esper (2003)	Argentina	https://pesquisa.bvsalud.org/portal/resource/pt/lil-349312	8
Mota et al. (2014)	Minas Gerais, Brazil	doi: 10.1590/1413-81232014197.09312013.	4
Nobre et al. (2016)	Brazil	doi: 10.1007/s40368-016-0248-6.	3
Peres et al. (2012)	Pelotas, Brazil	doi: 10.1902/jop.2011.110250.	4
Rapp et al. (2001)	Bahia, Brazil	https://pubmed.ncbi.nlm.nih.gov/12666945/	3
Nascimento A, et al. (2022)	Brazil	https://www.ncbi.nlm.nih.gov/pmc/articles/PMC9568304/	4
Rebelo et al. (2009)	Manaus, AM, Brazil	doi: 10.1590/s1806-83242009000300005.	2
Rojo Botello et al. (2011)	Mexico	https://www.scielo.org.mx/scielo.php?script=sci_arttext&pid=S1870-199X2011000100006	3
Sabogal et al. (2019)	Peru	doi: 10.1155/2019/2357013	3
Santosh et al. (2020)	Caribbean. Jamaica, Dominican Republic, and Puerto Rico.	doi: 10.1177/0272684X19895901.	4
Segundo et al. (2004)	Contagem, Minas Gerais, Brazil	doi: 10.1590/s0102-311x2004000200029.	3
Silva; Maltz (2001)	Porto Alegre, Brazil	https://pubmed.ncbi.nlm.nih.gov/11705268/	4
Silva-Boghossian et al. (2011)	Brazil	https://pubmed.ncbi.nlm.nih.gov/22068186/	3
Souza; Taba Jr. (2004)	Brazil	doi: 10.1590/s0103-64402004000100009.	3
Strauss et al. (2009)	Chile	doi.org/10.1186/s12903-019-0975-2	6
Susin; Albandar (2005)	Brazil, Porto Alegre	doi: 10.1902/jop.2005.76.3.468	2
Teixeira et al. (2019)	Sao Paulo, Brazil	https://doi.org/10.6084/m9.figshare.11314157.v1	3
Teixeira et al. (20200	Sao Paulo, Brazil	doi: 10.1590/1807-3107bor-2020.vol34.0058	3
Tinoco EM et al. (1997)	Brazil	doi: 10.1111/j.1600-0722.1997.tb00174.x.	2

The studies described in Table 1 are not included in the “references” section. These studies were excluded in the selection process. This table was kept in the manuscript to ensure transparency for the reader.

Reasons for exclusion: 1 - Reviews; 2 - Unclear or unsuitable case definition; 3 - Convenience sample; 4 - Lack prevalence data; 5 - Children population < 12-year-old; 6 - Same data from other study; 7 - Unrelated; 8 - Full text not accessed.

### Study characteristics

**Figure 2:**
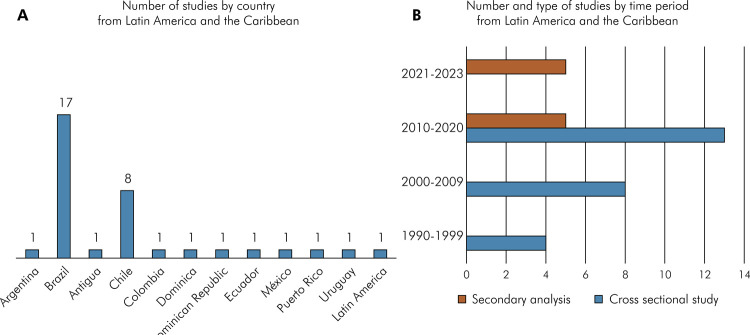
Number of studies by country (a) and by time period and type of studies (b) from Latin America and the Caribbean, found in the search strategy included in the review.

**Figure 3:**
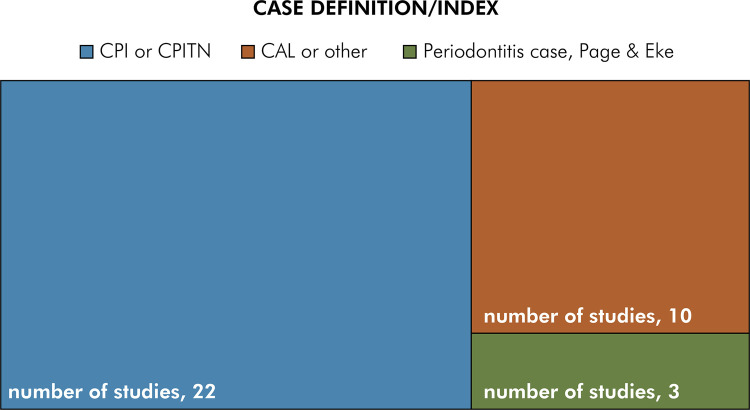
Number of studies according to case definition or index used as periodontal criteria.

These 35 studies analyzed populations from 12 of the 33 countries in Latin America and the Caribbean (36.3%), with Chile and Brazil reporting more than one study (Figure 2a). From the 1990s, the number of studies increased, particularly studies reporting secondary analysis of previously published data (Figure 2b). Regarding their methodology, diverse periodontal examination protocols and case definitions were used. The majority of studies (n=22) used the CPI index as a case definition (62.9%), three studies (8.6%) used the case definition proposed by Page and Eke^12^ for the surveillance of periodontal diseases, and ten studies (28.5%) used other case definitions, including CAL and gingival inflammation (Figure 3). All primary studies described sample size calculation, random selection of participants, examiner training, and funding sources.

### Periodontitis prevalence in nationally representative studies

Nationally representative studies were reported in four countries (Table 2): Brazil, Uruguay, Chile, and Colombia.

**Table 2:** Description of included studies for periodontal disease in adults.

Authors (year)	Country, City	Inclusion criteria	Exclusion criteria	Type of studied	Sample size (n)	Periodontal examination protocol	Age interval (years)	Periodontal criteria	Prevalence (CI) (%)	Prevalence by sex (%) female/male
Gamonal et al. (1998)^13^	Chile, Santiago	Adults, 35–44 and 65–74 year-old	NR	Cross sectional, randomly	868 adults and 217 adult seniors	Ten index teeth, CPITN index, WHO probe	35–44 65–74	CPITN = 3 CPITN = 4 CPITN = 3 CPITN = 4	50.2 40.6 28.5 71.4	NR
Susin et al. (2004)^14^	Brazil, Porto Alegre	Adults, 30 years and older	Edentolous and participant diagnosed with psychiatric problems or intoxicated with alcohol or drugs	Cross sectional, randomly	853	Six sites per tooth in full- mouth, PCP10-SE periodontal probe	30–103	Cases were defined as individuals with ≥ 30% teeth with CAL ≥ 5 mm	49.7	40.5/54.9
Susin et al. (2005)^15^	Brazil, Porto Alegre	Adults, 30 years and older	Edentolous, individuals diagnosed with serious psychiatric problems, or were intoxicated with alcohol or drugs	Cross sectional, randomly	853	Six sites per tooth in full- mouth, PCP10-SE periodontal probe	30–103	At least one tooth with PD:		
								PD ≥ 4 mm PD ≥ 5 mm PD ≥ 6 mm PD ≥ 7 mm	79.6 65.2 35.3 25.4	72.2/87.8 55.6/75.9 27.6/43.8 20.3/31.0
Lorenzo et al. (2005)^16^	Uruguay	Adults and elderly	Edentolous	NRS. Cross sectional, First National Survey of Oral Health 2010-2011	adults = 358 and elderly 411	Six index teeth, CPI index, WHO probe	35–44 and 65–74	Periodontal disease was defined as:		
								Moderate to severe when CPI > 2 and CAL ≥ 4 mm	21.8	12.9/30.1
								Severe when CPI > 2 and CAL ≥ 6	9.1	6.5/1.7
Borges-Yáñez et al. (2006)^17^	México, three different populations in Central Mexico	Adults, 60 years and older of urban areas of middle and low income level, and a rural area	Edentolous	Cross sectional, randomly	365	Two sites per tooth in partial-mouth, Michigan periodontal probe	60 and older	At least two sites with CAL 4 mm or more	50.7	NR
Romanelli et al. (2007)^18^	Argentina	Adults, 18–84 years of age, who spontaneously attended general dental services, with at least two teeth in any sextant	Patients with risk of endocarditis, diabetes or i mmunologic disease, and patients receiving corticoids or i mmunosuppressor drugs	Cross sectional	3,694	Six sites per tooth in full- mouth, CPI index, WHO periodontal controlled pressure probe, Sensor Probe Type C	18–84	CPI = 1 CPI = 2 CPI = 3 CPI = 4	17.2 38.8 26.4 14.3	NR
Corraini et al. (2008)^44^	Brazil, microarea Cajaíba	Adolescents and adults, ≥ 12 year-old	Edentolous	Cross sectional, census	194	Six sites per tooth in full- mouth, PCP-UNC-15, periodontal probe	12–82	At least one site with CAL or PD by age:		NR
								20-29 CAL ≥ 5 mm CAL ≥ 7 mm PD ≥ 5 mm PD ≥ 7 mm 30–39 CAL ≥ 5 mm CAL ≥ 7 mm PD ≥ 5 mm PD ≥ 7 mm 40–49 CAL ≥ 5 mm CAL ≥ 7 mm PD ≥ 5 mm PD ≥ 7 mm ≥ 50 CAL ≥ 5 mm CAL ≥ 7 mm PD ≥ 5 mm PD ≥ 7 mm	37.1 8.1 30.1 4.8 70.0 20.0 37.5 10.0 83.3 66.7 70.8 29.2 100 83.3 60.0 20.0	
								At least one site with CAL:		
Gamonal et al. (2010)^19^	Chile	Adults, urban, aged 35 to 44 year-old and adult seniors aged 65–74 year-old	Edentolous	Cross sectional, randomly	1,092 adults and 469 adult seniors	Six sites per tooth in full- mouth, PCP-UNC-15, periodontal probe	35–44	CAL > 3 mm CAL > 4 mm CAL > 5 mm CAL > 6 mm	93.4 77.5 58.3 38.6	92.3/94.9, 72.6/836 41.6/66.5, 32.3/46.4
								At least one site with CAL.		
							65–74	CAL > 3 mm CAL > 4 mm CAL > 5 mm CAL > 6 mm	97.5 92.7 81.4 69.3	97.3/98.0, 90.9/95.4 76.7/88.2, 62.6/79.1
Frias et al. (2011)^20^	Brazil, Guarulhos	Adults, 35–44 year-old	NR	Cross sectional, randomly	263		35–44	CPI = 0 CPI = 1 CPI = 2 CPI = 3 CPI = 4	10.6 (7.3–14.8) 9.1 (6.1–13.1) 53.6 (47.6–59.9) 22.4 (17.7–27.8) 4.2 (2.2–7.2)	NR
								At least one site with CAL:		
Susin et al. (2011)^45^	Brazil, Porto Alegre	Adolescents and young adults, 14–29 year-old	Subjects with serious neurologic or psychiatric conditions were excluded and Aggressive periodontitis cases	Secondary study from a larger sample representative of the population of Porto Alegre	584 (174, 20-24 year-old and 154, 25-29 year-old)	Six sites per tooth in full- mouth, PCP10-SE periodontal probe	20–24	CAL ≥ 3 mm CAL ≥ 4 mm CAL ≥ 5 mm CAL ≥ 6 mm	53.4 (43.0–63.8) 35.4 (24.2–46.6) 17.2 (9.5–25.0) 9.5 (3.3–15.8)	NR
								Chronic periodontitis was defined as CAL ≥3 mm affecting two or more teeth.	43.5 (32.0–55.0)	NR
								At least one site with CAL:		
							25–29	CAL ≥ 3 mm CAL ≥ 4 mm CAL ≥ 5 mm CAL ≥ 6 mm	79.4 (69.5–89.3) 56.4 (44.1–68.7) 29.0 (24.3–33.6) 13.8 (6.6–20.9)	NR
								Chronic periodontitis was defined as CAL ≥3 mm affecting two or more teeth.	72.0 (57.4–86.6)	NR
								At least one site with CAL or PD:		
Gaio et al. (2012)^21^	Brazil, Porto Alegre	Elderly, 60 years and older	Presence of conditions that may pose health risks, or that may interfere with the clinical examination	Cross sectional, randomly, subsample	217	Six sites per tooth in full- mouth, PCP10-SE periodontal probe	≥ 60	CAL ≥ 4 mm CAL ≥ 5 mm CAL ≥ 6 mm CAL ≥ 7 mm PD ≥ 4 mm PD ≥ 5 mm PD ≥ 6 mm PD ≥ 7 mm	100 94.1 84.4 73.9 74.5 62.6 31.8 22.2	NR
Bonfim et al. (2013)^22^	Brazil, Southern region	Adults in urban area, 35–44 year-old	Edentulism, refusal to participate, being bedridden, inability to answer the questions	Secondary study from a larger sample representative of the population	743	CPI index, WHO probe	35–44	CPI = 0 CPI = 1 CPI = 2 CPI = 3 CPI = 4	36.5 2.0 47.1 9.5 2.1	NR
Vettore et al. (2013)^23^	Brazil	Adults, 35–44 year-old	Without complete data	NRS. Secondary study from Brazilian Oral Health Survey 2010	4,594	Six ndex teeth, CPI index and CAL, WHO probe	35–44	Moderate-to-severe (CPI > 2 and CAL ≥ 4 mm)	15.3	13.5/14.9
								Severe periodontal disease (CPI > 2 and CAL ≥ 6 mm)	5.8	3.6/5.7
Giacaman et al. (2016)^46^	Chile, Maule region	Population from Maule region urban and rural, 15, 35–44 and 65–74 year-old	NR	Cross sectional, randomly	2,414	Six index teeth, CPI index, WHO probe	35–44 and 65–74	CPI = 1 CPI = 2 CPI = 3 CPI = 4 CPI = 1 CPI = 2 CPI = 3 CPI = 4	2.9 77.2 17.2 2.5 1.5 65.8 25.0 3.8	2.9/3.1, 77.3/76.6 16.9/18.8, 2.6/1.6 2.1/0.0, 68.7/56.9 23.6/29.2, 3.1/6.2
Schuch et al. (2018)^24^	Brazil, Pelotas	Adults, 31 year-old	NR	Secondary study from the 1982 Pelotas Birth Cohort Study	539	Six sites per tooth in full- mouth, PCP2 periodontal probe with 2- mm banding	31	CDC-AAP (2012) case definition periodontitis		
								Any periodontitis	37.3	31.6/42.9
								Moderate-to-severe	14.3	10.1/18.3
Serrano Suarez (2019)^25^	Colombia	Adults, 18 years and older	Presence of uncontrolled diseases, severe physical or mental disability, and health conditions that would require antibiotic. Edentolous, >79 year-old	NRS. Cross sectional, randomly	9,255	Six sites per tooth in full- mouth, PCP-UNC-15, periodontal probe	18–79	CDC-AAP (2012) case definition periodontitis		
								Mild	7.3	7.8/6.2
								Moderate	43.6	42.0/45.3
								Severe	10.6	7.5/13.9
Arantes et al. (2021)^26^	Brazil, Central-West	Adults, 35–44 year-old non-Indigenous	NR	NRS. Secondary study from Brazilian Oral Health Survey 2010	1,83	Six ndex teeth, CPI index and CAL, WHO probe	35–44	CPI = 2 CPI = 3 + CPI = 4	43.9 (37.4-50.7) 30.5 (28.2-32.9)	NR
Hugo et al. (2022)^27^	Brazil	NR	NR	Secondary study from Global Burden Disease 2019	NR	NR	NR	CPITN = 4 or CAL > 6 mm or PD > 5 mm	11.9 (8.6-15.4)	NR
Morales et al. (2022)^47^	Chile	Adults, urban, aged 35–44 and 65–74 year-old	Edentolous	NRS. Secondary study from First Chilean National Examination Survey 2007-2008	1,456 adults	Six sites per tooth in full- mouth, PCP-UNC-15, periodontal probe	35–44 and 65–74	CDC-AAP (2012) case definition		NR
								Mild	1.4	
								Moderate	57.2	
								Severe	29.7	
								AAP/EPP (2018) stage of periodontitis		
								Stage I	0.1	
								Stage II	4.7	
								Stage III	12.8	
								Stage IV	81.3	
Filgueiras et al. (2023)^28^	Brazil	Adults users of public services, 35–44 year-old	Edentolous and insufficient dental sextants for CAL examination	NRS. Secondary study from Brazilian Oral Health Survey 2010	3,426	Six ndex teeth, CPI index and CAL, WHO probe	35–44	At least two sites with CAL > 3 mm, and at least one site with PD > 3 mm, not necessarily at the same site	14.5	NR
León et al. (2023)^29^	Chile	Elderly, 65 years and older	NR	Secondary study from Global Burden Disease 2019	NR	NR	65–69 70–74 75–79 80–84 85–89 90–94 > 94	CPI = 4 (probing score > 5.5 mm)	29.1 (22.0–37-5) 26.8 (20.2-34.9) 25.4 (19.2–32.1) 24.4 (18.1–30.7) 23.8 (16.9–30.3) 23.2 (15.9–30.0) 22.7 (14.7–30.8)	NR

CPITN: community periodontal index treatment needs; CAL: clinical attachment loss; PD: probing deep; CPI: community periodontal index; NRS: National representative study; NR: Not reported data.

In Brazil, two studies analyzed data from the Brazilian Oral Health Survey 2010 for ages 35–44 years-old.^23,28^ These studies examined six index teeth, and the CPI and CAL case definitions were used. Vettore et al.^23^ reported a prevalence of 15.3% for moderate-to-severe periodontitis (CPI > 2 and CAL ≥ 4 mm) and 5.8% for severe periodontitis (CPI > 2 and CAL ≥ 6 mm). For both levels of severity of periodontitis, men showed a higher prevalence. Filgueiras et al.^28^ reported that 14.5% of the people analyzed had at least two sites with CAL > 3 mm and at least one site with PD >3 mm, not necessarily at the same periodontal site.

In Uruguay, using the same methodology as Vettore et al., Lorenzo et al.^16^ analyzed data from the First National Survey of Oral Health 2010-2011. For ages 35-44 and 65– 74 years-old together, a prevalence of 21.8% and 9.1% for moderate-to-severe and severe periodontitis, respectively., were reported. In particular, men had a higher prevalence of moderate-to-severe periodontitis, and women had a higher prevalence of severe periodontitis.

In Chile, Gamonal et al.^19^ analyzed data from the First Chilean National Examination Survey 2007-2008, which was carried out using a full-mouth evaluation of six periodontal sites per tooth. For the ages of 35–44 years-old, 93.4% and 38.6% of individuals had at least one periodontal site with CAL > 3 mm or CAL > 6 mm, respectively. For 65–74 years old, 97.5 and 69.3% of individuals had at least one periodontal site with CAL > 3 mm or CAL > 6 mm, respectively. When a secondary analysis of these data was performed, combining both age groups, Morales et al.^47^ reported a periodontitis prevalence of 88.3% (1.4% for mild periodontitis, 57.2% for moderate periodontitis, and 29.7 for severe periodontitis) using the Page and Eke^12^ case definition. Using the classification proposed by the AAP-EFP,^48^ a prevalence of 98.9% was reported, and most individuals were classified as stage IV periodontitis (81.3%).

In Colombia, Serrano and Suarez^25^ analyzed the Colombian Oral Health Study 2014 data, in which people ≥ 18 years-old received full-mouth evaluation of six periodontal sites per tooth. Using the case definition proposed by Page and Eke,^12^ the prevalence of periodontitis was 61.5% (7.3% for mild periodontitis, 43.6% for moderate periodontitis, and 10.6% for severe periodontitis). Among men, the prevalence of severe periodontitis was higher (7.5% versus 13.9%).

### Periodontitis prevalence in the adult population

For adults, studies analyzing the periodontitis prevalence were reported in six countries (Table 2): Argentina,^18^ Brazil,^14,15,20-24,26-28,44,45^ Chile,^13,19,29,46,47^ Colombia,^25^ México,^17^ and Uruguay.^16^

Different results were obtained in these studies depending on the age group analyzed and the case definition used. In fact, when periodontitis was defined as having at least one periodontal site with PD > 3 mm or CPI > 2, periodontitis prevalence varied between 11.6% and 99.9%. In contrast, when periodontitis was defined as having at least one periodontal site with CAL ≥ 5 mm, Susin et al.^45^ reported in young adults from Porto Alegre, Brazil, a periodontitis prevalence of 17.2% in ages 20–24 years-old and 29.0% in 25–29 years-old was reported. For adults aged ≥ 60 years-old from Porto Alegre, Gaio et al.^21^ reported a periodontitis prevalence of 94.1%. In the same country , for adults in Cajaíba, Corraini et al.^44^ reported a periodontitis prevalence of 37.1% in the 20–29 years-old group, which increased to 70.0%, 83.3%, and 100% in the age groups 30–39, 40–49, and ≥ 50 years-old, respectively. With the same case definition, Gamonal et al.^19^ reported a periodontitis prevalence of 58.3% and 81.4% in Chilean age groups 35-44 and 65-74 years-old, respectively. In these studies, the periodontitis prevalence was lower when a stricter case definition was used, for instance, more than one tooth with CAL or the combination of PD and CAL. Indeed, periodontitis prevalence varied between 14.5% and 72.0% for moderate-to-severe periodontitis^16,17,23,25,28,45^ and between 5.8% and 49.7% for severe periodontitis.^14,16,23^ In general, all these studies reported a higher prevalence of periodontitis in men.

### Gingivitis and periodontitis prevalence in the adolescent population

To report the prevalence of gingivitis and periodontitis in adolescents, studies were conducted in seven countries (Table 3): Antigua and Barbuda,^31^ Brazil,^35,37,38,40,42,44,45^ Chile,^32,33,36,46^ Dominica,^30^ Ecuador,^43^ Dominican Republic,^34^ and Puerto Rico.^41^ In addition, a multicenter study. was conducted, in which adolescents from Argentina, Chile, Colombia, Ecuador, and Uruguay were analyzed.^39^

**Table 3:** Description of included studies for periodontal disease in adolescents.

Authors (year)	Country, City	Inclusion criteria	Exclusion criteria	Type of studied	Sample size (n)	Periodontal examination protocol	Age interval (years)	Periodontal criteria	Prevalence (CI) (%)	Prevalence by sex (%) female/male
Leake et al. (1990)^30^	Dominica	Children, 12 year-old, attending scholl	NR	Cross sectional, randomly	332	Six index teeth, CPITN index, WHO probe	12	CPITN = 0 CPITN = 1 + CPITN = 2	17	NR
									62	
Vignarajah (1994)^31^	Caribbean Island Antigua y Barbuda	Children and adolescents, attending urban and rural schools, 12, 15–19 year-old	NR	Cross sectional, randomly	246, and 456	Six index teeth, CPITN index, WHO probe	12	CPITN = 0 CPITN = 1 CPITN = 2	26.0 28.0 43.0	NR
							15–19	CPITN = 0 CPITN = 1 CPITN = 2 CPITN = 3 CPITN = 4	14.0 13.0 56.0 14.0 3.0	
Lopez et al. (1996)^32^	Chile, Santiago	Adolescents, 15–19 year-old attending high school		Cross sectional, randomly	2,4	Six index teeth, CPITN index, WHO probe	15–19	CPITN = 0 CPITN = 1 CPITN = 2 CPITN = 3 CPITN = 4	5.4 14.8 62.2 9.5 0.9	NR
Lopez et al. (2001)^33^	Chile, Province Santiago	Adolescents, 12–21 year-old attending high school	Not be examined due to constraints such a trismus	Cross sectional, randomly	9,162	Six sites of first and second molars and incisors	12–14	At least one site with CAL ≥ 3 mm	2.5	2.2/2.9
							15–17		3.7	4.7/2.8
							18–21		6.8	5.2/7.9
Collins et al. (2005)^34^	Dominican Republic, Santo Domingo	Adolescents, 12–21 year-old attending high school	No CAL detected	Cross sectional, randomly	1,963	Six sites of first and second molars and incisors	12–21	At least one site with		
								CAL ≥ 1 mm CAL ≥ 2 mm CAL ≥ 3 mm	49.5 15.0 4.0	49.3/49.6 15.1/14.9 4.2/3.7
Corraini et al. (2008)^44^	Brazil, microarea Cajaíba	Adolescents and adults, ≥ 12 year-old	Edentolous	Cross sectional, census	194	Six sites per tooth in full- mouth, PCP-UNC-15, periodontal probe	12–82	At least one site with CAL or PD by age: 12-19		NR
								CAL ≥ 5 mm CAL ≥ 7 mm PD ≥5 mm PD ≥7 mm	7.7 5.1 5.1 5.1	
Antunes et al. (2008)^35^	Brazil, Sao Paulo	Adolescents, 15–19 year-old	Asian and Amerindian categories	Cross sectional, randomly	1,799	Six index teeth, CPI index, WHO probe	15–19	CPI = 0 CPI = 1 CPI = 2	65,7 (63.5-67.9) 21.6 (19.7-23.6) 19.4 (17.6-21.3)	NR 19.5/24.5 17.4/22.1
Susin et al. (2011)^45^	Brazil, Porto Alegre	Adolescents and young adults, 14–29 year-old	Subjects with serious neurologic or psychiatric conditions were excluded and Aggressive periodontitis cases	Secondary study from a larger sample representative of the population of Porto Alegre	584	Six sites per tooth in full- mouth, PCP10-SE periodontal probe	14–19	At least one site with CAL:		
					(256 14–19 year-old)			CAL ≥ 3 mm CAL ≥ 4 mm CAL ≥ 5 mm CAL ≥ 6 mm	22.3 (12.2–32.5) 7.4 (2.0–12.8) 2.5 (0.4–4.6) 18.2 (7.9–28.4)	NR NR NR NR
								Chronic periodontitis was defined as CAL ≥ 3 mm affecting two or more teeth.		NR
Wauters et al. (2014)^36^	Chile, Castro	Children aged 12 year-old attending urban, public and private-subsidized schools	Students with fixed orthodontics appliances and/or a pathology, such as Down syndrome, trismus and epilepsy	Cross sectional, randomly	242	Six index teeth, CPITN index, WHO probe	12	CPITN = 0 CPITN = 1 CPITN = 2 CPITN = 3	0.0 42.2 44.2 13.6	0.0/0.0 45.0/38.9 42.6/46.0 12.4/15.0
Leão et al. (2015)^37^	Brazil, Caiuá, São Paulo	Adolescents, 10–19 year-old, rural school	Refused study participation	Cross sectional, census	180	Six index teeth, CPI index, WHO probe	10–19	CPI = 1 CPI = 2 CPI = 3	77.7 20.8 1.5	NR
	State									
Fonseca (2015)^38^	Brazil, Vale do Jequitinhonha	Adolescents, 15–19 year-old	Individuals with difficulties cognitive or mentalis	Cross sectional, randomly	450	CPI index with some modifications	15–19	CPI = 0 CPI = 1 CPI = 2	3.5 51.5 8.4	3.3/3.8 57.8/44.2 6.6/10.5
Morales et al. (2015)^39^	Latin America, Capital cities from countries in South America, Argentina, Chile, Colombia, Ecuador and Uruguay	Adolescents, 15–19 year-old attending public and private high school	Subjects undergoing fixed orthodontic treatments or with any condition that required antibiotic prior to the periodontal examination	Cross sectional, randomly, multicenter	1,07	Six sites per tooth in full- mouth, PCP-UNC-15, periodontal probe	15–19	At least one site with CAL or PD:		
								CAL ≥ 3 mm PD ≥ 4 mm BoP ≥ 25%	32.6 59.3 28.6	35.8/29.1 58.9/59.6 34.1/22.7
Tomazoni et al. (2016)^40^	Brazil, Santa Maria	Children aged 12 year-old attending public schools	NR	Cross sectional, randomly	1,134	Six index teeth, CPI index, WHO probe	12	Gingivitis was considered if:		NR
								At least one surface showed CPI = 1	96.2 (95.1–97.3)	
								Using a cut-off point of > 15% bleeding	26.2 (23.7–28.8)	
Giacaman et al. (2016)^46^	Chile, Maule region	Population from Maule region urban and rural, 15, 35–44 and 65–74 year-old	NR	Cross sectional, randomly	2,414	Six index teeth, CPI index, WHO probe	15	CPI = 1 CPI = 2 CPI = 3 CPI = 4	8.7 74.4 16.3 0.0	11.0/6.8 71.1/77.0 17.0/15.8 0.0/0.0
Elias-Boneta et al. (2018)^41^	Puerto Rico	Children, 12 year-old, attending public and private schools, physical status ASA I and ASA II	Participants with conditions requiring antibiotic prophylaxis	Cross sectional, randomly	1,586	Gentle probing into gingival sulcus of the buccal surface, PCP UNC 126 periodontal probe	12	At least one site presented gingival bleeding.	80.4	79.5/81.2
								Gingivitis limited: 2–4 teeth or 25%–49% of the teeth examined presented gingival bleeding	19.5	18.7/20.4
								Extensive gingivitis: > 5 teeth or > 50% of the teeth examined presented gingival bleeding	60.8	60.8/60.8
Knack et al. (2019)^42^	Brazil	Adolescents, 12, 15–19 year-old	NR	NRS. Secondary study from Brazilian Oral Health Survey 2010	12,773 (7,328 12-year-old and 5,445 15–19)	Six ndex teeth, CPI index and CAL, WHO probe	12, 15–19	CPI = 1 CPI = 2 CPI = 3 CPI = 4	32.0 33.1 4.5 0.3	31.8/32.2 31.6/34.6 NR NR
Michel-Crosato et al. (2019)^43^	Ecuador, Quito	Children aged 12 year-old attending public and urban schools	NR	Cross sectional, randomly	1,1	Six index teeth, CPIT index, WHO probe	12	CPITN = 1	92.0	NR
Morales et al. (2022)^47^	Capital cities from countries in South America, Argentina, Chile, Colombia, Ecuador and Uruguay	Adolescents from South America, attending schools, 15–19 year-old	Edentolous	Secondary study from a sample of adolescents from different countries in South America 2010-2012	1,07	Six sites per tooth in full- mouth, PCP-UNC-15, periodontal probe	15–19	CDC-AAP (2012) case definition		
								Mild Moderate Severe	11.4 15.3 0.5	NR
								AAP/EPP (2018) stage of periodontitis		
								Stage I Stage II Stage III Stage IV	39.3 28.2 7.6 0.5	NR

CPITN: community periodontal index treatment needs; CAL: clinical attachment loss; PD: probing deep; CPI: community periodontal index; BoP: bleeding on probing; NRS: national representative study; NR: not reported data.

In the case of periodontitis, when the case definition involved the CPI, a prevalence not exceeding 16.3% was observed (adolescents with at least one periodontal site with PD > 3 mm or CPI > 2). In the multicenter study, a prevalence of 59.3% was reported. In contrast, when CAL was involved in the case definition, prevalences not exceeding 22.3% (adolescents with at least one periodontal site with CAL ≥ 3 mm) and 7.7% (adolescents with at least a periodontal site with CAL ≥ 5 mm) were observed. In the multicenter study, a prevalence of 32.6% for cases with CAL ≥ 3 mm was reported. Conversely, a lower prevalence was observed when a stricter case definition was used. Indeed, Susin et al.^45^ reported a prevalence of 18.2% (adolescents with more than one tooth with CAL ≥ 3 mm), and Morales et al.^47^ reported a prevalence of 15.3% (adolescents with detectable interdental CAL in at least two non-adjacent teeth), with 8.1% of individuals classified as stage III or IV periodontitis (adolescents with at least two non-adjacent teeth with interdental CAL ≥ 5 mm).

In the case of gingivitis, a high prevalence was reported when the gingivitis case was established as the detection of gingival bleeding and at least one local factor (such as dental calculus) in at least one periodontal site (CPI = 1 or CPI = 2). In particular, the gingivitis prevalence values were 28% in Antigua y Barbuda, 62% in Dominica, 80.4% in Puerto Rico, and 92% in Ecuador ^30,31,41,43^. In Chile, the prevalence of gingivitis ranged between 8.7% and 42.2% in the different cities studied.^32,36,46^ In Brazil, the gingivitis prevalence ranged between 21.6% and 96.2% in the cities studied;^35,37,38,40^ in a national study conducted with 12 year-old adolescents and those between 15-19 years old, a prevalence of 33.1%.^42^ was reported

### Results reanalysis using CPI as the case definition

**Figure 4:**
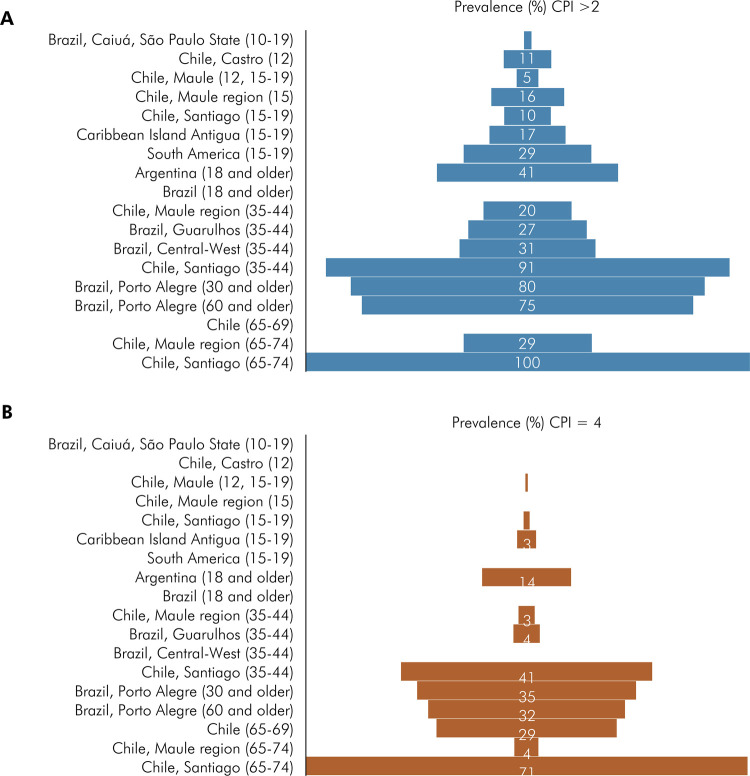
Studies categorized according to prevalence (%) using CPI index criteria, (a) CPI > 2 and (b) CPI = 4, ordered by age.

To perform a comprehensive analysis of the findings summarized herein, the data from 18 studies (51%) from 4 countries and the multicenter study in adolescents were re-categorized as CPI > 2 and CPI = 4. Then, periodontal disease prevalences were ordered according to age (Figure 4). Two studies did not provide data to establish the category CPI >2,^27,29^ and two other studies for CPI =4.^26,39^ The data reanalysis revealed that for adolescents, the prevalence of periodontal disease with CPI > 2 ranged between 2% to 29%, and with CPI = 4 did not exceed 3%. However, prevalence increased considerably with age, reaching 99.9% in the most affected population (65–74 years-old, in Chile), with 71.4% of individuals being classified as CPI = 4.

### Comprehensive analysis of the studies that used the Page y Eke recommended case definition

**Figure 5:**
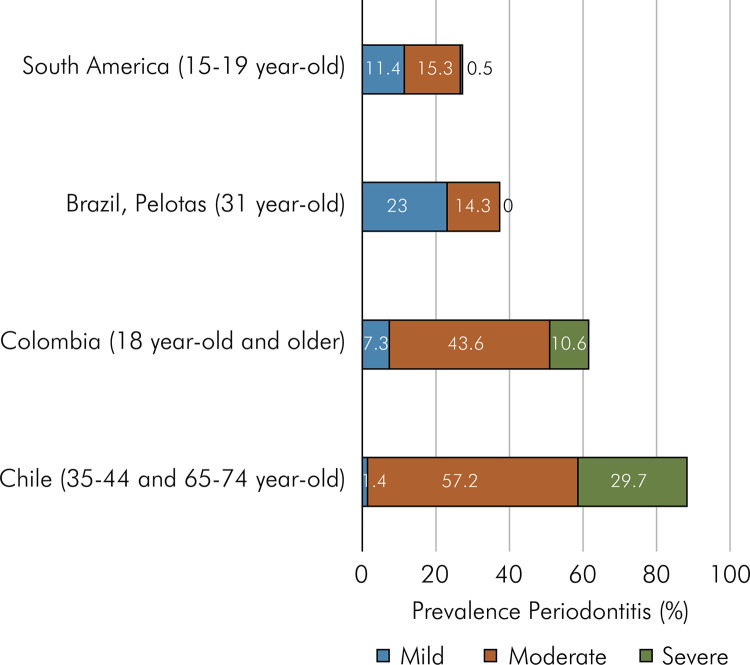
Stacked bar charts showing the prevalence of periodontitis in adolescents or adults according to the studies in Latin-American that used CDC/AAP case definition.

Figure 5 shows the prevalence of periodontitis in the four studies that used the case definition recommended by Page and Eke^12^. In the multicenter study with adolescents, a prevalence of 27.2% of periodontitis was reported, with 0.5% of subjects having severe periodontitis.^47^ In Pelotas, Brazil, subjects a t the age of 31 years showed a prevalence of moderate-to-severe periodontitis of 37.3% and a prevalence of severe periodontitis of 14.3% was reported.^24^ Then, in the national study carried out in Colombia, in the age range of 18 to 79 years old the periodontitis prevalence was 61.5%, with 10.6% of subjects having severe periodontitis.^25^ Finally, in the national study conducted in Chile,, for subjects aged between 35–44 and 65–74 years old,, the prevalence of periodontitis was 88.3%, with 29.7% of subjects having severe periodontitis.^47^ To sum up these results confirmed that with increasing age, the prevalence of periodontitis also increases.

## Discussion

In the present study, an updated review of the epidemiological studies that have analyzed the prevalence of periodontal disease in adolescents and adults living in Latin America and the Caribbean was carried out. According to our findings, the situation described by Botero and Oppermann in 2015^6,7^ was maintained at the end of 2023. Although the number of regional or nationally representative studies has increased, the methods used and the case definition were found to be heterogeneous. Furthermore, the evidence available was insufficient to describe the region globally. Indeed, most countries have no nationally representative epidemiological studies; when they did have, the majority of them were approximately 30 years old. Nevertheless, we could conclude that periodontal disease significantly affected the vast majority of the populations analyzed, and its prevalence increased significantly with age, showing a prevalence ranging between 15.3% to 59.3% in adolescents and 11.6% to 99.9% in adults.

Nationally representative studies of the adult population have been conducted in Brazil, Colombia, Chile, and Uruguay. In the studies from Brazil and Uruguay, a prevalence values of 5.8% and 9.1% severe periodontitis were reported in 35–44 year old adults, when the case definition used involved the CPI index and the CAL with partial mouth registration. Similarly, in the study from Colombia, a prevalence of severe periodontitis of 10.6% was reported at the age of 18–79 years, using the case definition proposed by Page and Eke with full-mouth registration. However, in Chile, a higher prevalence of severe periodontitis was reported, reaching 29.7% at the combined age group of 35–44 and 65–74 years old, also using the Page and Eke proposal of case definition with full-mouth registration. Therefore it was evident that the different prevalence values of severe periodontitis reported among these countries were, at least partly, due to the different ages of the individuals analyzed and the various methods of periodontal evaluation. Moreover, it is noteworthy that multiple social determinants were identified., including income, which have a meaningful impact on the increase in the prevalence of periodontal diseases.^49,50^ Indeed, as previously established, partial records may underestimate the prevalence of periodontitis.^51^ Furthermore, it was also evident that the variability of results were due to the heterogeneous definition of the periodontal case.

A criterion widely used to define severe periodontitis is PD > 6 mm, given its relevance in public health and because it contributes to determining the need for periodontal treatment. Recently, this case definition was used in the Global Burden of Disease study conducted by Wu et al.,^4^ and a prevalence of severe periodontitis in adults of around 19% was reported for the America continent. In the present review, when the articles using the case definition of PD > 6 mm or a CPI = 4 were analyzed, a prevalence between 14% and 71% was observed, depending on the age of the Latin American adults analyzed. This higher prevalence of severe periodontitis in Latin America could be related to a greater frequency of social determinants that contribute to the burden of chronic non-communicable diseases, such as periodontitis, and include low socioeconomic and education levels and high prevalence of risk factors. Particularly in Chile, the prevalence of smoking and type II diabetes mellitus is higher than that of other Latin American countries, and it could contribute to its higher prevalence of severe periodontitis.^52-55^

Concerning the current classification of periodontal and peri-implant diseases and conditions proposed by the AAP/EFP, it should be considered with caution when used in studies to describe the prevalence of periodontitis. In the study by Morales et al., two primary studies were re-analyzed, and the individuals were re-classified considering the case definition proposed by Page and Eke^12^ and the current AAP/EFP classification.^48^ In the first case, the prevalence of severe periodontitis was 0.5% in adolescents and 29.7% in adults. Conversely, in the second case, the prevalence was much higher, reporting that 8.1% of adolescents and 94.1% of adults had stage III and IV periodontitis. Therefore, as has been established elsewhere, the classification proposed by the AAP/EFP is not recommended for use in epidemiological studies since it tends to overestimate the prevalence of periodontitis and, consequently, the need for periodontal treatment.^56,57^

When the studies conducted in different Latin America and the Caribbean countries were comprehensively analyzed, a high prevalence of gingivitis in adolescents was revealed. Therefore,, a challenge was generated to identify and resolve this disease early and stop its progression to periodontitis. In the same way, although with less evidence, a high prevalence of periodontitis was revealed in adults, which could lead to severe forms of the disease that can compromise the general health and quality of life of individuals. One of the limitations of the present review was that a search of the gray literature was not carried out, nor were databases from universities and ministries of health analyzed. Thus, different studies on the prevalence of gingivitis or periodontitis in the region that could have been helpful for our analysis were not considered. Nevertheless, one of the strengths of our study was the systematization of all the literature published in the traditional databases tb means of a broad search without language and time restrictions. This allowed an update of the knowledge as from December 2023 and complemented with the articles that were reported for the region until 2015.^6,7^ Although few articles met the inclusion criteria established in this review, they informed us about active Latin American and Caribbean countries at the level of university campuses with studies in specific populations that provided relevant information, which undoubtedly contributed to local decision-making on health issues. Indeed together, these studies showed that the prevalence of periodontal disease increases with the age of the population, which is a critical determinant that must be considered when defining public policies on periodontal health. This is particularly relevant when, in recent years, an accelerated aging process has been evident in Latin America and the Caribbean.^8,58^ Moreover, emerging evidence establishes that chronological aging and premature periodontal immunosenescence contribute to the pathogenesis of periodontitis.^59,60^

In this context, the efforts of various professional and scientific organizations and societies are notable, which have handled their resources to reveal periodontal health problems worldwide, including the Global Report of the WHO, the World Dental Federation (FDI), the International Association for Dental Research (IADR), the European Organization for Caries Research/European Federation of Periodontology (ORCA/EFP) Consensus, and the Latin American Oral Health Association (LAOHA) Consensus.^61^ Based on the findings herein, we recommend generating alliances and international consensus to adequately monitor gingivitis and periodontitis in the region. In particular, it is essential to agree on the definition of the periodontal case, standardized measurement criteria, establish homogenous evaluation methods, and age groups to analyze, as priorities for future epidemiological studies. In the last consensus convened by the LAOHA in 2015,^61^ the need to implement actions to promote prevention, professional education programs, early diagnosis, and timely treatment of periodontitis was identified. At this moment, we recommend designing and implementing multicenter studies with national representation in which a unique case definition is assessed, such as the CPI index or that proposed by Page and Eke. In this way, more significant information about the need for treatment (scaling and root plan) and estimation of the resources needed to address epidemiological studies would be obtained.^11,12,62^ Indeed, in such a way this needs to be done in such a way that it generates reliable, reproducible, and comparable data. The goal is to facilitate the organization and systematization of information to foster the generation of public policies, preventive plans, and early diagnosis and treatment strategies that allow us to resolve the serious periodontal reality in Latin America and the Caribbean.
